# Molecular Insights into the Action Mechanism, Resistance Development, and Ecological Risks of Cyantraniliprole

**DOI:** 10.3390/ijms27062897

**Published:** 2026-03-23

**Authors:** Jiabao Wu, Xiaohui Liu, Yuqing Peng, Jiguang Huang, Lijuan Zhou

**Affiliations:** State Key Laboratory of Green Pesticides, South China Agricultural University, Guangzhou 510642, China

**Keywords:** cyantraniliprole, ryanodine receptor, resistance, ecotoxicology, environmental fate, molecular mechanism

## Abstract

Cyantraniliprole, a second-generation diamide insecticide, exhibits broad-spectrum efficacy against numerous insect pests due to its selective activation of insect ryanodine receptors (RyRs). This activation triggers uncontrolled calcium release from the sarcoplasmic reticulum, resulting in sustained muscle contraction, paralysis, and ultimately death. Its unique mode of action, which is different from that of organophosphates, carbamates, pyrethroids, and neonicotinoids, helps minimize cross-resistance, making it a valuable component of integrated pest management (IPM). However, continuous field use has led to the development of resistance, primarily mediated by target-site mutations within the RyR transmembrane domain (e.g., *G4946E*, *I4743M*, and *I4790K*) and by enhanced metabolic detoxification via cytochrome P450 monooxygenases, carboxylesterases, and glutathione S-transferases. These mechanisms often confer cross-resistance to other diamide insecticides, thereby complicating resistance management. Moreover, sublethal exposures can disrupt insect growth, development, and reproduction, potentially accelerating resistance evolution. In addition, cyantraniliprole poses ecological risks due to its toxicity to non-target organisms such as aquatic species, including zebrafish and water fleas, pollinators such as honeybees, and soil fauna, as well as the environmental persistence of its major metabolite, J9Z38. This review comprehensively integrated current knowledge on the molecular mechanisms of action, genetic and metabolic bases of resistance, sublethal effects, and ecotoxicological impacts of cyantraniliprole, along with its environmental fate, plant uptake and translocation, and residue dynamics in agricultural systems. Finally, we discuss potential risk-mitigation strategies, including formulation optimization, application-method improvements, and resistance monitoring. Overall, this review aims to provide a comprehensive scientific foundation for the sustainable use, resistance management, and regulatory assessment of this widely used insecticide.

## 1. Background, Chemical Structure, Synthesis, and Mechanism of Action of Cyantraniliprole

### 1.1. Background and Chemical Structure

Cyantraniliprole, developed by DuPont, is a second-generation anthranilic diamide insecticide discovered after chlorantraniliprole. It exhibits favorable systemic properties and can be translocated through the xylem in plants. Cyantraniliprole possesses a broad spectrum and is effective against Lepidoptera (e.g., *Spodoptera litura* (Fabricius), *Spodoptera exigua* (Hübner), *Chilo suppressalis* (Walker)), Diptera (e.g., *Liriomyza sativae* Blanchard), Hemiptera (e.g., *Aphis gossypii* Glover, *Bemisia tabaci* (Gennadius)), Coleoptera (e.g., *Phyllotreta striolata* (Fabricius)), etc. It is particularly effective against the cotton aphid (*A. gossypii*) and the tobacco cutworm (*S. litura*) [1,2,3,4]. Currently, registered formulations such as oil-dispersion suspensions, suspension concentrates, and seed treatment suspensions are widely applied for pest management in crops including cabbage, pepper, tomato, cucumber, cotton, and rice [5,6].

Cyantraniliprole acts by activating insect ryanodine receptors (RyRs), triggering the uncontrolled release of calcium ions. This results in continuous muscle contraction, paralysis, and ultimately death of the insects [7]. This unique mechanism of action confers no cross-resistance with major insecticide classes, such as organophosphates, carbamates, pyrethroids, or neonicotinoids [8,9]. Additionally, cyantraniliprole shows high selectivity towards non-target arthropods and demonstrates favorable environmental compatibility, highlighting its strong potential for integrated pest management [10].

**Figure 1:**
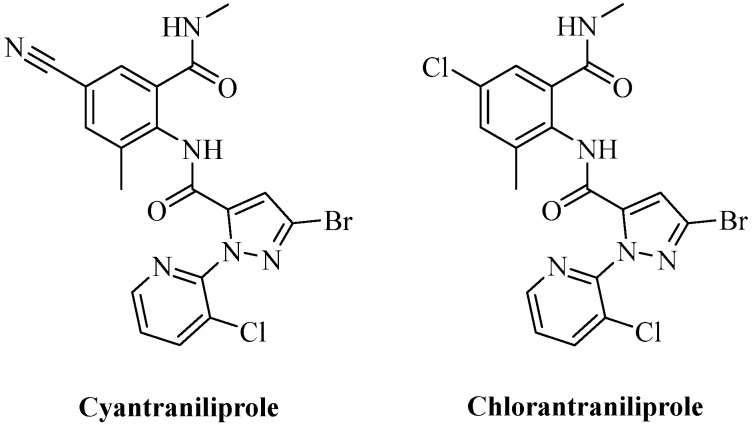
Structures of cyantraniliprole and chlorantraniliprole.

A key structural difference of cyantraniliprole from chlorantraniliprole is the introduction of a polar pyridine ring on the benzene moiety (Figure 1). This modification broadens its insecticidal spectrum, particularly enhancing its efficacy against piercing–sucking pests such as aphids and whiteflies. Furthermore, it improves the systemic mobility of the compound within plants [11,12,13,14,15,16]. Consequently, cyantraniliprole is generally considered to possess improved insecticidal activity, enhanced systemic properties, and a potentially reduced risk of resistance development, making it a promising alternative to chlorantraniliprole [12].

Among the diamide insecticides in IRAC Group 28, cyantraniliprole possesses a unique structural feature, in which a chlorine atom is replaced by a cyano group. This modification enhances its systemic properties and broadens its insecticidal spectrum. In addition to its high efficacy against lepidopteran pests, which are the primary targets of other diamides, cyantraniliprole is also effective against piercing–sucking insects such as aphids and whiteflies. As a result, it has wider applicability and greater representativeness for resistance monitoring of different pest types.

### 1.2. Synthetic Methods of Cyantraniliprole

**Figure 2:**
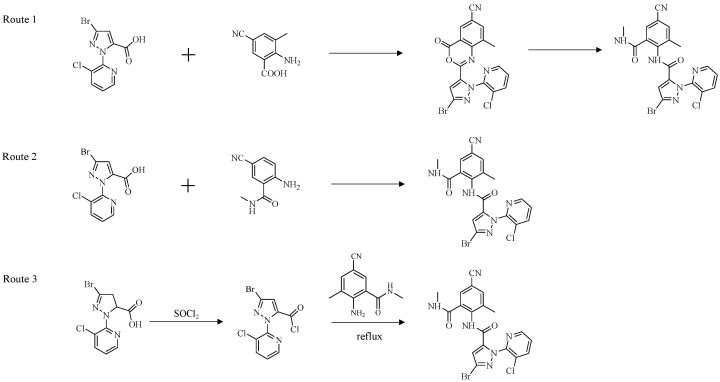
Major synthetic routes for cyantraniliprole. Original illustration by the authors, based on data from references [17,18,19,22].

Several synthetic routes have been reported for the production of cyantraniliprole, as outlined in Figure 2.

Route 1 involves the ring-opening reaction of 3-bromo-1-(6-chloro-2-pyridinyl)-N-(4-cyano-2-methyl-6-oxocyclohexa-1,4-dien-1-yl)-1H-pyrazole-5-carboxamide with methylamine. This approach is characterized by short reaction times and operational simplicity. However, the preparation of the key intermediate, 3-bromo-1-(6-chloro-2-pyridinyl)-N-(4-cyano-2-methyl-6-oxocyclohexa-1,4-dien-1-yl)-1H-pyrazole-5-carboxamide, remains challenging. Both the coupling of a pyrazolecarboxylic acid with an anthranilic acid and the reaction of a pyrazolecarbonyl chloride with an isatoic anhydride are relatively cumbersome and time consuming [17,18,19].

Route 2 proceeds via the condensation of a pyrazolecarboxylic acid with an anthranilamide, using methanesulfonyl chloride as a coupling agent in the presence of an acid acceptor [20].

Route 3 starts from 3-bromo-1-(3-chloro-2-pyridinyl)-4,5-dihydro-1H-pyrazole-5-carboxylic acid, which undergoes simultaneous acyl chlorination and oxidation of the pyrazoline ring to a pyrazole ring upon treatment with a suitable halogenating reagent. This integrated procedure provides the intermediate 3-bromo-1-(3-chloro-2-pyridinyl)-1H-pyrazole-5-carbonyl chloride, which is then coupled with a substituted aniline to give cyantraniliprole in high yield, without requiring an additional acid acceptor [21].

**Figure 3:**
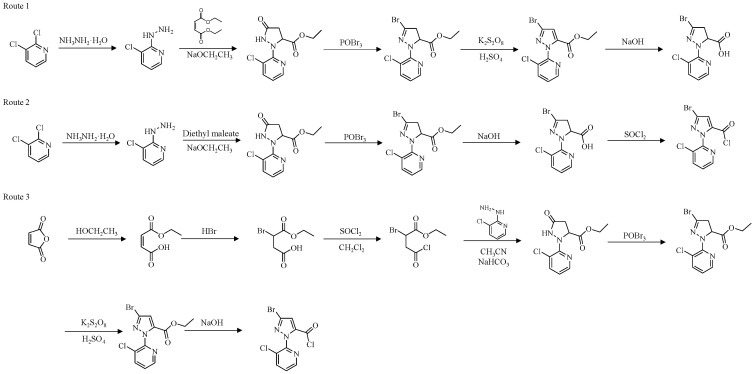
Synthetic routes to key intermediates. Original illustration by the authors, based on data from references [21,22,23].

The synthesis of key intermediates follows several routes (Figure 3):

### 1.3. The Ryanodine Receptor (RyR): Target and Mechanism of Action of Cyantraniliprole

The differential expression of RyR isoforms between insects and mammals provides the selectivity of diamide insecticides. In mammals, three functionally different RyR isoforms (RyR1, RyR2, and RyR3) are expressed in tissues such as skeletal and cardiac muscle [24,25,26,27,28]. In contrast, insects possess only a single RyR isoform, which is ubiquitously expressed in tissues, including muscle and nerve cells [29]. This exclusive reliance on a single isoform makes the insect RyR a highly specific molecular target for diamide insecticides [30,31,32,33].

The RyR is the largest known ligand-gated calcium-release channel. Its primary role is to regulate the efflux of calcium ions from intracellular stores, a process essential for key physiological functions such as muscle contraction, neurotransmitter release, hormone secretion, and cell proliferation [34,35]. Diamide insecticides, including cyantraniliprole, act by selectively and persistently activating the insect RyR.

## 2. Resistance: Mechanisms and Management Challenges

The development of insect resistance to cyantraniliprole has become a major threat to its sustainable use. Currently recognized resistance mechanisms primarily involve target site modifications and enhanced metabolic detoxification. Although field resistance to cyantraniliprole remains relatively limited, its long-term efficacy is increasingly threatened by the gradual evolution of resistance in pest populations. Mutations in the RyR gene represent a principal mechanism conferring cross-resistance within the diamide insecticide class. Target-site resistance mainly arises from amino acid substitutions at key residues within the RyR transmembrane domain—primarily *G4946E*, *I4743M*, and *I4790K*. These mutations alter the conformation of the binding pocket, directly impairing insecticide–target interactions. This mechanism not only leads to high-level resistance but also accounts for the cross-resistance observed among different diamide compounds. In addition to target-site resistance, metabolic resistance constitutes another major mechanism. It involves the overexpression or enhanced activity of detoxification enzymes, including cytochrome P450 monooxygenases (P450s), carboxylesterases (CarEs), and glutathione S-transferases (GSTs). The key genes involved (e.g., *CYP6CX3*, *mGST1*, *SlCOE030*) exhibit diverse regulatory patterns in different insect species. Furthermore, the discovery of non-metabolic genes such as *SfNrf6* has expanded our understanding of the complexity underlying cyantraniliprole resistance.

### 2.1. Cross-Resistance Mediated by Target-Site Mutations

It was reported that specific mutations in RyR could confer cross-resistance, although the degree of resistance might vary in different insecticides. For instance, in *Drosophila suzukii* Matsumura, resistance-conferring RyR mutations might affect the binding of chlorantraniliprole more significantly than that of cyantraniliprole [36]. In *Spodoptera frugiperda*, the *I4790K* mutation in the *RyR* gene conferred a high level of resistance to cyantraniliprole (resistance ratio up to 3414-fold) and simultaneously led to high-level cross-resistance to flubendiamide, chlorantraniliprole, and cyclaniliprole [37]. Further, it was confirmed that an *S. frugiperda* strain (Chlorant-R) selected with chlorantraniliprole developed cross-resistance to cyantraniliprole (RR ≈ 27-fold), although this level was considerably lower than its resistance to chlorantraniliprole itself (237-fold) [36]. These findings highlighted the inherent risk of cross-resistance among diamide insecticides, emphasizing that different chemical subclasses of diamides (e.g., anthranilic vs. phthalic diamides) or even compounds within the same subclass (e.g., chlorantraniliprole and cyantraniliprole) could not be simply regarded as rotation insecticides free of cross-resistance in resistance management strategies [36,38].

Specific mutations in RyR have been reported to confer cross-resistance among diamide insecticides, though the resulting resistance levels can vary with compounds. For example, in *D. suzukii*, resistance-conferring *RyR* mutations appeared to impair chlorantraniliprole binding more severely than cyantraniliprole binding [36]. In *S. frugiperda*, the *I4790K* mutation in the *RyR* gene conferred high-level resistance to cyantraniliprole (resistance ratio up to 3414 fold) and simultaneously induced pronounced cross-resistance to flubendiamide, chlorantraniliprole, and cyclaniliprole [37]. Furthermore, a chlorantraniliprole-selected strain of *S. frugiperda* (Chlorant-R) exhibited cross-resistance to cyantraniliprole (RR ≈ 27 fold), albeit at a markedly lower level than its resistance to chlorantraniliprole itself (237 fold) [36].

In various insect species, mutations in the transmembrane region of the *RyR* have been widely reported to be associated with resistance. Troczka et al. identified a glycine-to-glutamate mutation (*G4946E*) within a highly conserved transmembrane domain near the C-terminus of the *RyR* in resistant populations of *Plutella xylostella* (Linnaeus), which was closely associated with diamide resistance [39]. Based on the cloning of the full-length *RyR* cDNA sequence from *P. xylostella*, Guo et al. identified the same mutation site in resistant populations from two geographically different regions and functionally validated its role in resistance development [40]. In the same year, three additional RyR mutation sites (*E1338D*, *Q4594L*, and *I4790M*) associated with chlorantraniliprole resistance were identified in field populations of *P. xylostella* from Yunnan Province, China [41,42]. Homology modeling and molecular docking analyses revealed that the mutation of glycine to glutamate at position 4891 in the RyR of *S. frugiperda* disrupted key chemical interactions, reduced binding affinity, and consequently decreased the receptor’s sensitivity to diamide insecticides [43].

Furthermore, a nationwide monitoring program (2023–2025) targeting the major rice pest *Cnaphalocrocis medinalis* Guenée highlighted the critical role of target-site mutations. The study, covering 37 populations in eight major rice-growing regions in China, revealed severe resistance to chlorantraniliprole, along with the identification of two RyR mutations, *I4712M* and *Y4621C*. Of these, the *I4712M* mutation was fixed (100% frequency) in the highly resistant populations and showed a strong positive correlation with chlorantraniliprole resistance levels, confirming it as a key target site mutation responsible for high-level resistance [12].

Beyond target-site mutations, the regulation of gene expression represents another key mechanism inducing insecticide resistance. In *P. xylostella*, miR-7a and miR-8519 were shown to modulate the expression of *PxRyR*. Under such miRNA-mediated regulation, RyR expression was significantly elevated in field-resistant populations compared to susceptible ones, implicating miRNAs in the development of chlorantraniliprole resistance [20]. Similarly, Zhang et al. reported that in *S. frugiperda*, miRNA-190-5p down-regulated *CYP6K2* expression by binding to its 3′-UTR. Conversely, inhibiting this miRNA increased *CYP6K2* levels and enhanced chlorantraniliprole tolerance [44]. Furthermore, it was also demonstrated that Lnc-GSTu1-AS could maintain the stability of GSTu1 mRNA by blocking miR-8525-5p-mediated degradation of GSTu1, thereby enhancing resistance to chlorantraniliprole in *P. xylostella* [45].

### 2.2. Molecular Mechanisms of Target-Site Resistance

**Figure 4:**
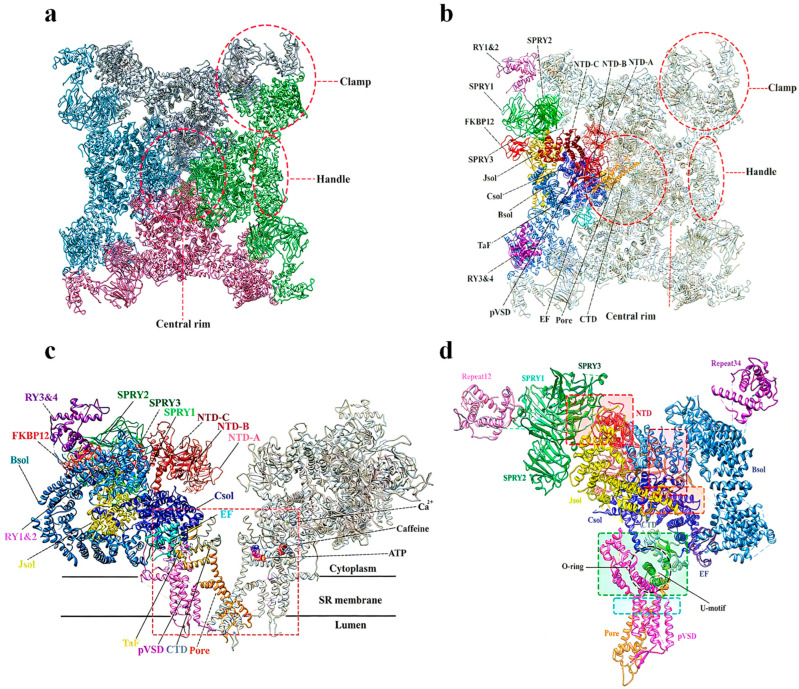
Overall structure and domain organization of RyR [47]. (**a**) The top view of the cyo-EM structure of a rabbit RyR1. The four protomers are colored in green, pink, cyan, and gray, respectively. Several distinct areas of the cytoplasmic region, including the clamp, the handle, and the central rim, are also labeled. (**b**) The top view of the cryo-EM structure of the rabbit RyR1 in a complex with FKBP12, Ca^2+^, ATP, and caffeine. (**c**) The side view of the cryo-EM structure of the rabbit RyR1 in a complex with FKBP12, Ca^2+^, ATP, and caffeine. (**d**) The locations of different ligand-binding sites within the full-length RyR1 are indicated by colored boxes. This figure was adapted from Hadiatullah H. et al., (2022) [47]. Structural Insight Into Ryanodine Receptor Channelopathies. Frontiers in Pharmacology, 13, 897494. https://doi.org/10.3389/fphar.2022.897494, under the terms of the Creative Commons Attribution (CC BY) license.

A precise understanding of the structural architecture of the RyR is essential for elucidating corresponding diamide-insecticide resistance mechanisms. FKBP12, a small immunophilin, functions as a key allosteric stabilizing regulator of RyR. Notably, FKBP12 and FKBP12.6 bind preferentially to RyR1 and RyR2, respectively, stabilizing their closed conformational states [46]. The top view of the cryo-EM structure of rabbit RyR1 (Figure 4a,b) [47]. The overall architecture of RyR1 was resolved by cryo-EM analysis of its tetramer in complex with FKBP12 (Figure 4c), while a detailed view of its domain organization is presented in Figure 4d [47,48].

The structure of RyR provided a structural basis for elucidating the molecular mechanisms of target-site mutation-mediated resistance in field and laboratory insect populations. While a direct high-resolution structure is not yet available, substantial evidence supports the conclusion that diverse diamide insecticides target a shared binding pocket.

**Figure 5:**
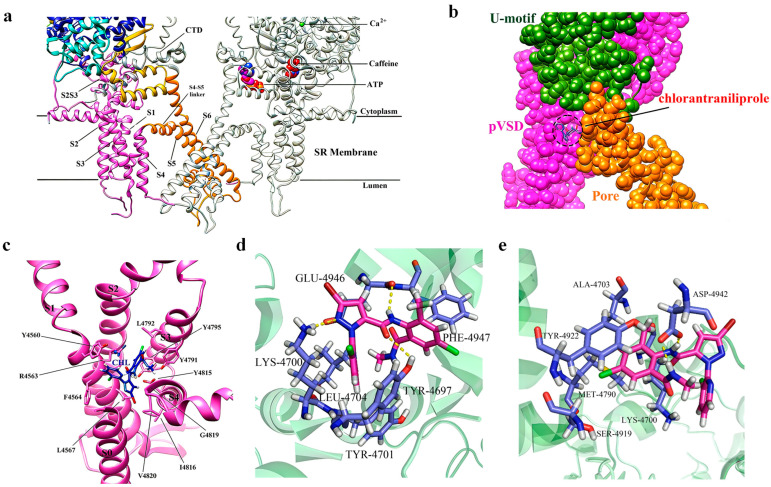
Structures of RyRs in complex with chlorantraniliprole [47,56]. (**a**) A close-up view of the binding sites of Ca^2+^, ATP, and caffeine, and the transmembrane region. (**b**) The zoomed-in views of the binding sites for chlorantraniliprole. (**c**) Zoomed-in view of the chlorantraniliprole binding site in RyR1. (**d**) Docking analyses between chlorantraniliprole and *P. xylostella RyRs-G4946E.* (**e**) Docking analyses between chlorantraniliprole and *P. xylostella RyRs-I4790M*. (**a**–**c**) were adapted from Hadiatullah H. et al., (2022) [47]. Structural Insight Into Ryanodine Receptor Channelopathies. Frontiers in Pharmacology, 13, 897494. https://doi.org/10.3389/fphar.2022.897494, under the terms of the Creative Commons Attribution (CC BY) license. (**d**,**e**) were adapted from Ren J Z et al., (2023) [56]. 3D-QSAR-Based molecular design to discover ultrahigh active *N*-phenylpyrazoles as insecticide candidates. Journal of Agricultural and Food Chemistry, 71, 4258−4271. https://doi.org/10.1021/acs.jafc.2c08719, used under license granted by Copyright Clearance Center.

Four specific mutations in the insect RyR transmembrane domain have been linked to resistance [48,49,50,51,52,53]. Resistance to diamide insecticides has evolved in multiple insect species via mutations in this transmembrane region. Key substitutions—*G4819E*, *C4657M*, *F4564D*, and *Y4795F* (corresponding to *G4946E*, *I4790M*, *Y4701D*, and *Y4922F* in *P. xylostella*)—reduced chlorantraniliprole affinity by 100- to 2000-fold [48,49,50,51,52]. Functional studies have established that a single mutation could confer high-level cross-resistance to multiple diamide insecticides, including cyantraniliprole, chlorantraniliprole, and flubendiamide [1,38]. This is further evidenced by recent functional genetics research, in which CRISPR/Cas9-mediated homozygous introduction of the *I4734M* mutation in *SfRyR* in *S. frugiperda* conferred high-level resistance to chlorantraniliprole (396.7-fold) and tetraniliprole (149.1-fold), alongside moderate-level resistance to cyantraniliprole (32.3-fold) and flubendiamide (29.5-fold) [54]. The most direct and compelling evidence for the mechanism of resistance has been provided by high-resolution structural elucidation of the ligand–receptor complex. The cryo-EM structure of rabbit RyR1 in complex with chlorantraniliprole, resolved by the Yuchi team, reveals that diamide insecticides bind specifically within the pore and voltage-sensing-like (pVSD) domain of the RyR transmembrane region (Figure 5). This binding is mediated by key interactions with residues such as Arg4563 and Asp4815 [47,55]. The binding mode of chlorantraniliprole with the *P. xylostella* RyR involves being surrounded by amino acid residues, including LEU4704, ASP4942, TYR4918, TYR4922, SER4919, ILE4790, LYS4700, and ALA4703, through hydrophobic and hydrogen-bond interactions. As shown in Figure 5d,e, compared with the normal *P. xylostella* RyR, the binding ability of chlorantraniliprole to *P. xylostella* RyR-I4790M was weakened due to the lack of a hydrogen bond formed with LYS4700. The binding position of chlorantraniliprole to *P. xylostella* RyR-G4946E shifted, which reduced its insecticidal activity. The binding energies of chlorantraniliprole to *P. xylostella* RyR-G4946E and *P. xylostella* RyR-I4790M were 8.277 and 7.454 kcal/mol, respectively [56].

Chlorantraniliprole and cyantraniliprole share the same binding site on the RyR but differ in their interaction modes. The chlorine atom in chlorantraniliprole mediates primarily hydrophobic interactions, whereas the cyano group in cyantraniliprole enables both hydrophobic and hydrogen-bonding interactions. This structural difference may explain their distinct activity profiles against various pest species.

Based on the molecular mechanisms elucidated above, practical recommendations for resistance management can be proposed. The shared binding pocket across diamide insecticides explains their clear cross-resistance [57]. Different diamide subclasses (e.g., cyantraniliprole and chlorantraniliprole) should not be used in rotation. Monitoring should focus on key resistance mutations such as *G4946E* (or *G4900E*) and *I4743M*.

### 2.3. Metabolic Resistance

Metabolic resistance represents a primary mechanism by which insects develop resistance to insecticides, mainly characterized by enhanced detoxification capacity. The three key enzyme systems involved—cytochrome P450 monooxygenases (P450s), carboxylesterases (CarEs), and glutathione S-transferases (GSTs)—constitute the major detoxification families associated with insect metabolic resistance [58]. Elevated activity or overexpression of these enzymes commonly enhances metabolic detoxification, which also induces cross-resistance among insecticide classes [59,60,61,62]. In particular, increased P450 activity or expression is widely recognized as a major mechanism responsible for cross-resistance in different insecticide classes [61].

The central role of P450s in metabolic resistance has been confirmed in multiple insect species. In *B. tabaci*, populations resistant to cyantraniliprole showed significantly elevated P450 activity compared with susceptible strains. Molecular analyses further revealed obvious upregulation of the *CYP6CX3* gene in resistant populations. This gene is considered to metabolize cyantraniliprole through both dealkylation and hydroxylation pathways, representing a key metabolic resistance mechanism. In a highly resistant *B. tabaci* strain from Shanxi, China (SX-R), synergist bioassays demonstrated a pronounced synergistic effect of the P450 inhibitor piperonyl butoxide (PBO) on cyantraniliprole (synergistic ratio = 4.6), whereas inhibitors of glutathione S-transferases (DEM) and esterases (TPP) showed no significant synergism. These results directly indicate that enhanced P450 activity is the primary metabolic mechanism underlying high-level resistance to cyantraniliprole in this strain [63]. Similarly, in the silkworm *Bombyx mori* Linnaeus, exposure to cyantraniliprole significantly induced midgut CYP450 activity and upregulated several P450 genes [64]. This finding not only supports the involvement of P450s in cyantraniliprole metabolism but also implies the potential for P450-mediated cross-resistance to other insecticides.

In addition to P450s, other detoxification enzyme systems also contribute to resistance in various insect species and can lead to broad-spectrum cross-resistance. In *Bactrocera dorsalis* Hendel, resistance to cyantraniliprole was associated with elevated activities of P450s, CarEs, and GSTs [64]. Particularly, the resistant strain also exhibited moderate cross-resistance to spinosyns, organophosphates, and pyrethroids [65]. In *D. suzukii*, research has clearly demonstrated that the *mGST1* gene is a key detoxification gene responsible for cyantraniliprole resistance [36].

However, considerable variation in detoxification mechanisms exists among different insect species and populations. For instance, GST and CarE activities were not significantly elevated in some resistant populations of *B. tabaci* or in *B. mori* [64,66]; studies on the high-resistance *B. tabaci* strain from Shanxi also confirmed that GSTs and CarEs did not contribute substantially to its high-level resistance to cyantraniliprole [67]. These variations underscore the need for population-specific monitoring and mechanism identification when designing resistance management strategies that account for metabolic resistance.

A recent study in *S. litura* provides molecular insights into such species-specific detoxification mechanisms. Cytochrome P450 genes in *S. litura* are involved in the detoxification of cyantraniliprole. Treatment with cyantraniliprole significantly upregulates a P450 gene (*SlCYP9A75a*) specifically expressed in the Malpighian tubules of *S. litura*. Through homology modeling and molecular docking analysis, it was confirmed that the *SlCYP9A75a* protein can bind to cyantraniliprole, and the residue formed a hydrogen bond with cyantraniliprole is PRO-467, with a bond distance of 2.8 Å. PRO-467 was identified as a key binding site for the interaction between the *SlCYP9A75a* protein and the insecticide cyantraniliprole [68].

### 2.4. Other Mechanisms and Novel Gene Discovery

In addition to the three primary detoxification enzyme systems, other mechanisms can also confer cross-resistance or tolerance to cyantraniliprole. For instance, Li et al. demonstrated that the plant secondary metabolite, nicotine, specifically upregulates the gut-specific carboxylesterase gene *SlCOE030* in the midgut of *S. litura*. This gene encodes a carboxylesterase capable of metabolizing both nicotine and cyantraniliprole, resulting in cross-tolerance to these two compounds [2]. In *S. frugiperda*, upregulation of the *SfNrf6* gene has been associated with resistance to cyantraniliprole. This gene is highly expressed in the midgut and responds rapidly to insecticide stress. Knocking down *SfNrf6* via RNA interference (RNAi) significantly restored susceptibility to the insecticide, whereas its overexpression directly conferred tolerance. The expression level of *SfNrf6* may thus serve as a biomarker for early resistance monitoring [63].

**Figure 6:**
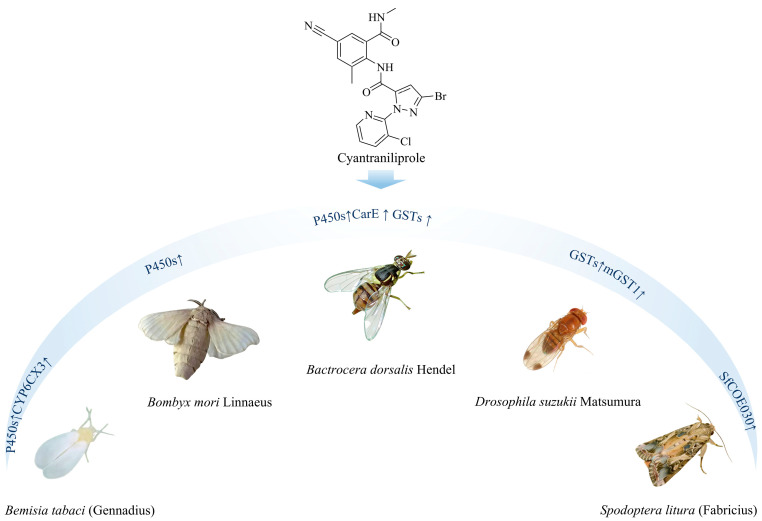
Molecular mechanisms of metabolic resistance to cyantraniliprole in different insect species. The arrow denotes upregulation of these genes upon cyantraniliprole treatment. Original illustration by the authors, based on data from references [36,58,59,60,61,62,63,64,65,66,67,68].

Research indicates that resistance to cyantraniliprole frequently confers cross-resistance to other diamide insecticides (e.g., chlorantraniliprole) and may extend to compounds with distinct modes of action. Therefore, resistance management strategies should carefully assess cross-resistance risks across insecticide classes. Rotation among compounds with shared target sites or detoxification pathways should be avoided, and monitoring of key resistance-related markers—such as RyR mutations, *P450* genes, and *SfNrf6* expression—should be strengthened in field populations. The molecular mechanisms of metabolic resistance to cyantraniliprole in different insect species are shown in Figure 6.

### 2.5. Potential Cross-Resistance with Structurally Related Compounds

Beyond target-site mutations and metabolic detoxification, another consideration for resistance management is the potential for cross-resistance with insecticides that share structural features or binding sites with cyantraniliprole. Although cyantraniliprole exhibits no cross-resistance with major insecticide classes, such as organophosphates, carbamates, pyrethroids, or neonicotinoids, due to its distinct mode of action [8,9], the situation is more complex for compounds containing pyrrole moieties.

Traditional pyrrole insecticides, such as chlorfenapyr, act on mitochondria by disrupting oxidative phosphorylation, a mechanism entirely different from RyR activation [69]. Therefore, no cross-resistance between cyantraniliprole and chlorfenapyr is expected.

However, a novel class of compounds, pyrrole-2-carboxamides, has recently been identified as potent RyR activators. These compounds exhibit sub-micromolar potency against insect RyRs, comparable to that of commercial diamide insecticides such as chlorantraniliprole. Radioligand-binding studies have demonstrated that pyrrole-2-carboxamides share a common binding domain with anthranilic diamides. Furthermore, the well-characterized target-site mutation *G4946E* in the RyR, which confers high-level resistance to diamide insecticides, also greatly reduces sensitivity to pyrrole-2-carboxamides. These findings indicate a shared binding site and the potential for cross-resistance between pyrrole-2-carboxamides and diamide insecticides, including cyantraniliprole. Although pyrrole-2-carboxamides currently lack strong insecticidal activity against key pest species in whole-insect screens [70], their discovery highlights the possibility that future compounds containing pyrrole structures could interact with the RyR. Therefore, resistance-monitoring programs should remain vigilant for potential cross-resistance if such compounds are developed for commercial use.

## 3. Sublethal Effects and Ecotoxicology

### 3.1. Impacts on Insect Populations

Following field application, insecticides may cause sublethal effects on a portion of the pest population due to differences in individual exposure levels and the gradual decline of insecticide efficacy over time. Such sublethal exposure can influence insect ecology, behavior, growth, development, and reproduction, and may further accelerate the evolution of insecticide resistance [71,72]. For example, in *S. frugiperda*, sublethal doses of cyantraniliprole suppressed larval growth (reducing body and pupal weight, delaying development) and impaired adult reproduction (reducing fecundity) [10]. These physiological inhibitions (reduced growth, survival, and reproduction) observed in the fall armyworm were consistent with the sublethal effects of cyantraniliprole on other major pests. Similar responses were reported in the beet armyworm (*S. exigua*) [73], tobacco cutworm (*S. litura*) [74], oriental tobacco budworm (*Heliothis assulta* Guenée) [75], and white-backed planthopper (*Sogatella furcifera* (Horvath)) [1]. This consistency observed in various species indicates that cyantraniliprole is likely to engage common physiological inhibitory mechanisms at sublethal concentrations.

At the metabolic level, these sublethal effects are largely driven by disruptions in energy homeostasis. Carbohydrate and lipid metabolism were the primary energy resources during insect growth and development [75,76]. Sublethal exposure to cyantraniliprole has been shown to obviously disrupt energy homeostasis in insects, especially in *S. frugiperda*. Key carbohydrate reserves—including glucose, trehalose, and glycogen—undergo significant depletion following treatment, together with a marked reduction in triglyceride levels, indicating concurrent impairment of lipid metabolism [77]. This broad suppression of energy substrates likely contributes to the observed delays in larval development and reduced pupal weight, and may diminish the physiological fitness of pests under insecticide stress. Such metabolic dysregulation not only explains part of the sublethal effects but also suggests that insects surviving sublethal exposure may encounter sustained physiological costs, potentially altering population dynamics and selection pressures in the field.

### 3.2. Toxicity to Non-Target Organisms

Owing to its environmental persistence, cyantraniliprole is frequently detected in surface water and sediments, where it is regarded as an emerging pollutant [78] with potential risks to aquatic ecosystems [79,80,81]. The insecticide exhibits high acute toxicity to several non-target organisms, including the water flea (*Daphnia magna* Straus), the silkworm (*B. mori*), and the Italian honey bee (*Apis mellifera ligustica* Spinola), which severely limits its application potential [82,83]. Moreover, its primary degradation product, J9Z38, shows even greater environmental persistence than the parent compound, with a half-life ranging from 77 to 220 days. Recognized as a toxicologically relevant transformation product, J9Z38 may contribute to long-term environmental contamination [84,85].

#### 3.2.1. Risks to Aquatic Ecosystems

Cyantraniliprole exhibits varying degrees of toxicity to multiple aquatic organisms. It shows high toxicity to the aquatic midge *Chironomus dilutus*, with the LC_50_ at 96 h being 4.7 μg/L—approximately 1.5-fold greater than that of imidacloprid [67,79]. For the red swamp crayfish (*Procambarus clarkii* Girard), the LC_50_ at 96 h is 149.77 mg/L; exposure induces abnormal behaviors such as hyperactivity and increased excretion, and tissue accumulation follows the order: gill > hepatopancreas > intestine > muscle, with the gills exhibiting the highest residue levels [67,74].

In fish, cyantraniliprole displays moderate acute toxicity to zebrafish (*Danio rerio*), with the LC_50_ values at 24 h and 96 h being 7.2 mg/L and 3.5 mg/L, respectively. Long-term sublethal exposure (0.35 mg/L, 30 days) leads to broad alterations in gene expression and metabolite profiles, including down-regulation of receptor-related genes, up-regulation of fatty-acid metabolism genes, and changes in metabolites such as taurine and betaine [86]. Integrated omics analyses further reveal that cyantraniliprole disrupts hepatic fatty-acid metabolism, inhibits ABC transporter function, and activates the PPAR-signaling pathway, triggering a detrimental cycle of toxin accumulation, metabolic dysregulation, and oxidative stress that ultimately disrupts liver homeostasis. Additionally, cyantraniliprole can induce apoptosis in the heart and axial muscle of zebrafish larvae, and its 30-day no-observed-effect concentration (NOEC) is 0.0157 mg/L, suggesting possible adaptive responses under chronic exposure [80,81,87].

For the tilapia (*Oreochromis mossambicus* Peters), cyantraniliprole demonstrates embryotoxicity and genotoxicity, inhibiting embryonic development and causing DNA damage [76]. After 28-day exposure of juveniles to concentrations ranging from 0.037 to 3.7 mg/L, specific growth rates decreased by 7–30% in a concentration-dependent manner, indicating that long-term, low-level exposure significantly impairs fish growth [88].

#### 3.2.2. Risk to Soil Ecosystems

Cyantraniliprole shows considerable persistence in soil, with a half-life ranging from 16 to 324 days [64,65], and poses toxic effects on non-target soil organisms. The compound is susceptible to hydrolysis and photolysis in the environment. Its major degradation product, J9Z38, exhibits even greater environmental persistence than the parent compound, with a half-life of 77–220 days, and is regarded as a toxicologically relevant compound capable of contributing to long-term environmental contamination [63,66]. Toxicity studies indicate that both cyantraniliprole and its primary metabolite, J9Z38, can induce oxidative stress in earthworms at concentrations as low as 5.0 mg/kg, ultimately resulting in cellular damage. In particular, cyantraniliprole itself produces a greater degree of oxidative injury compared to J9Z38 [66,67].

In addition to its effects on soil fauna, cyantraniliprole also significantly impacts soil microbial communities. At high concentrations (10 and 50 mg/kg), cyantraniliprole significantly inhibits the activities of dehydrogenase, acid phosphatase, and alkaline phosphatase. Acid phosphatase inhibition persists for 60 days, damaging the microbial community responsible for phosphorus mineralization, whereas urease activity recovers after only short-term fluctuations [89]. Under repeated application conditions, cyantraniliprole alters the soil microbial community structure, suppressing *Clostridium sensu stricto* 1 (involved in organic matter cycling) while enriching potentially degradative bacteria such as *Sphingomonas*, *Marmoricola*, and the *Burkholderia*-*Paraburkholderia* genus. It also inhibits urease and sucrase activities and disrupts key metabolic pathways related to nitrogen and carbon metabolism [90].

#### 3.2.3. Toxicity to Pollinators and Other Beneficial Arthropods

Cyantraniliprole posed toxicity risks to pollinator insects and other beneficial arthropods. It is highly toxic to pollinator insects such as the Italian honey bee (*A. mellifera ligustica*) [82,83]. The acute oral LD_50_ (48 h) of the 97% cyantraniliprole technical material for the Italian honey bee, the Chinese honey bee (*Apis cerana Cerana*), and the Carniolan honey bee (*Apis mellifera carnica* Pollman) ranged from 0.180 to 0.239 μg/bee [82]. These data highlight the urgent need to develop strategies that alleviate non-target impacts and improve application safety for this insecticide [91].

#### 3.2.4. Biochemical Mechanisms of Toxicity and Detoxification in Non-Target Organisms

Beyond insect systems, biochemical insights into the metabolism and detoxification of cyantraniliprole have also been gained from aquatic organisms and microbial communities.

In zebrafish exposed to cyantraniliprole and its major metabolite, J9Z38, increased reactive oxygen species (ROS) and malondialdehyde (MDA) levels disrupted cellular oxidative balance, leading to oxidative damage [87,92]. Superoxide dismutase (SOD) activity was significantly enhanced from day 7 onward in the cyantraniliprole treatment group, while SOD activity in the J9Z38 group was significantly higher than the control on days 14 and 28. Glutathione S-transferase (GST) activity increased in response to both compounds, indicating activation of antioxidant and detoxification enzyme systems [87]. The stronger and earlier effects induced by cyantraniliprole suggest that its metabolic transformation within the organism may be a critical detoxification process.

Transcriptomic analysis further revealed the molecular basis of this differential toxicity. Cyantraniliprole induced 11,729 differentially expressed genes (DEGs), significantly enriched in calcium-signaling pathways, whereas J9Z38 induced 3331 DEGs primarily enriched in basic metabolic pathways (e.g., amino acid, sugar, and lipid metabolism) and immune responses, reflecting a general stress response [87]. Molecular docking showed that cyantraniliprole forms both hydrogen bonds (with Tyr22, Arg254) and hydrophobic interactions (with Pro23, Pro48, etc.) with target proteins, whereas J9Z38 only engages in hydrophobic interactions (with Arg254, Cys26, Gln279). The stronger and more diverse binding of cyantraniliprole to RyR-related proteins in zebrafish may underlie its greater toxicity, and the loss of hydrogen-bonding capacity upon metabolism to J9Z38 likely contributes to its reduced toxicity.

In sediment environments, the metabolism and detoxification of cyantraniliprole and J9Z38 are primarily mediated by microbial community restructuring and methanogen-driven methane metabolism pathways. Both compounds reduced microbial diversity, but cyantraniliprole induced a more complex microbial co-occurrence network, indicating greater stress on the microbial community [87]. Key degrading populations were identified as methanogenic genera *Methanolinea*, *Methanoregula*, and *Methanosaeta*, whose abundances increased significantly upon exposure, participating in pollutant degradation via methane metabolism pathways [93,94]. Metabolomic analysis confirmed increased levels of seven metabolites typical of organic pollutant degradation and decreased levels of 25 lipids and lipid-like molecules, reflecting inhibition of microbial membrane structure and function [95,96]. Ten common differential metabolic pathways (primarily amino acid and fatty acid metabolism) were shared between the two compound treatments, confirming that structural analogs induce similar metabolic perturbations [97,98]. The less negative impact of J9Z38 on the microbial community compared to the parent compound further confirms that metabolism can, to some extent, reduce the environmental risk of cyantraniliprole [87].

In addition to sediment microorganisms, lactic acid bacteria (LAB) have recently been shown to possess the ability to degrade cyantraniliprole residues on food crops. Kiruthika et al., reported that *Lactobacillus pentosus* and *Lactococcus lactis* subsp. *L. lactis* removed more than 98% of cyantraniliprole within 4 days in nutrient broth, achieving complete degradation by day 10. In field trials on curry leaves, application of these LAB strains reduced cyantraniliprole residues by 40.99% and 34.52%, respectively, within 8 h of spraying. These findings highlight the potential of LAB as an eco-friendly strategy for reducing pesticide residues in food commodities [99].

### 3.3. Exposure and Risk Assessment in Rice–Crayfish Co-Culture Systems

In integrated rice–crayfish-farming systems, the application window of cyantraniliprole coincides with the peak activity period of red swamp crayfish (*P. clarkii*), raising concerns over non-target exposure [88,100,101,102,103,104]. Field studies confirm that while cyantraniliprole provides effective control of the rice stem borer (*C. suppressalis*), it exhibits a dynamic bioaccumulation profile in crayfish tissues, characterized by an initial increase followed by clearance within several weeks. Importantly, no significant adverse effects on crayfish growth or yield have been reported under recommended field doses. Residue analysis indicates that the maximum cyantraniliprole levels detected in crayfish remain well below the established health-based guidance values (e.g., Acceptable Daily Intake). Therefore, under standard agricultural practices, dietary exposure from consuming crayfish grown in such systems is considered negligible and complies with food-safety standards [105].

### 3.4. Risk to Human Health

In vitro studies using human liver cells (L-02) show that cyantraniliprole alone exhibits relatively low toxicity, with an EC_50_ of 217.4 μmol/L, considerably higher than that of emamectin benzoate (6.21 μmol/L). However, when combined with emamectin benzoate, a synergistic toxic effect is observed: cell viability decreases from 85.56% (4 μmol/L emamectin benzoate + 15 μmol/L cyantraniliprole) to 43.21% (5 μmol/L emamectin benzoate + 15 μmol/L cyantraniliprole). The mixture significantly elevates intracellular ROS and MDA levels, overwhelms antioxidant defenses, and disrupts glycerophospholipid and fatty acid metabolism pathways associated with oxidative stress [106]. Therefore, the synergistic toxicity of cyantraniliprole under mixed-exposure conditions should be noted.

## 4. Environmental Behavior and Residues

### 4.1. Uptake, Translocation, and Distribution in Plants

Cyantraniliprole exhibits systemic properties in various crops, yet its patterns of uptake, translocation, and distribution vary significantly among plant species. In general, its systemic movement depends primarily on xylem-mediated upward transport driven by transpiration, whereas phloem-mediated basipetal translocation is weak or absent.

In soybean, cyantraniliprole is absorbed by roots and translocated to floral tissues, and it displays greater mobility within leaves compared with chlorantraniliprole, showing faster uptake and translocation [107,108]. In maize, the compound exhibits limited bidirectional movement, with acropetal transport predominating. Uptake peaks at 24 h after treatment, and translocation involves an active, carrier-mediated process [10,108].

In wheat and rice, root uptake occurs mainly via the apoplastic pathway. In wheat, the insecticide accumulates predominantly in the soluble fraction of leaf cells after upward translocation [109], whereas in rice, translocation capacity is relatively limited—the root translocation factor (TF) remains below 1, and phloem-mediated basipetal movement is very weak [12].

In tomato, cyantraniliprole is transported only upward through the xylem and lacks phloem mobility. Its distribution is influenced by tissue position and transpiration rate, resulting in the highest accumulation in mature leaves and the lowest residues in fruits [110,111].

**Figure 7:**
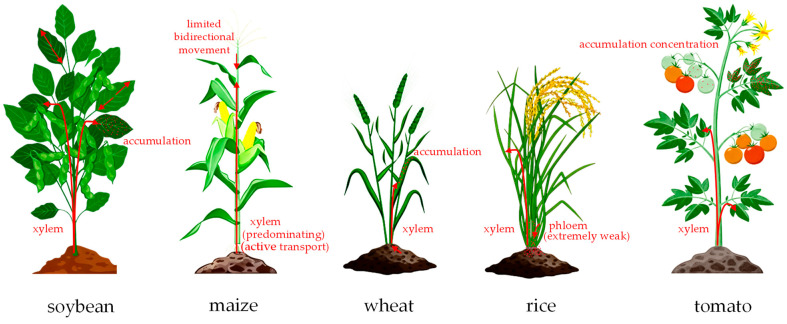
Translocation of cyantraniliprole in plants. Arrows indicate the direction of cyantraniliprole movement; red dots mark accumulation sites. Original illustration by the authors, based on data from references [10,12,108,109,110,111,112].

Key factors affecting the distribution of cyantraniliprole in plants include transpiration rate, tissue type and developmental stage, and compound-specific properties. Compared with chlorantraniliprole, cyantraniliprole shows faster uptake and translocation in certain crops, which may be related to differences in degradation and metabolism within plant tissues [112]. Overall, the residue distribution of cyantraniliprole in plants generally follows the sequence: older leaves > younger leaves > fruits, a pattern that has important implications for evaluating its efficacy persistence and environmental-residue risks. The translocation of cyantraniliprole in plants is illustrated in Figure 7.

### 4.2. Metabolic Pathways and Degradation Products in Crops

**Figure 8:**
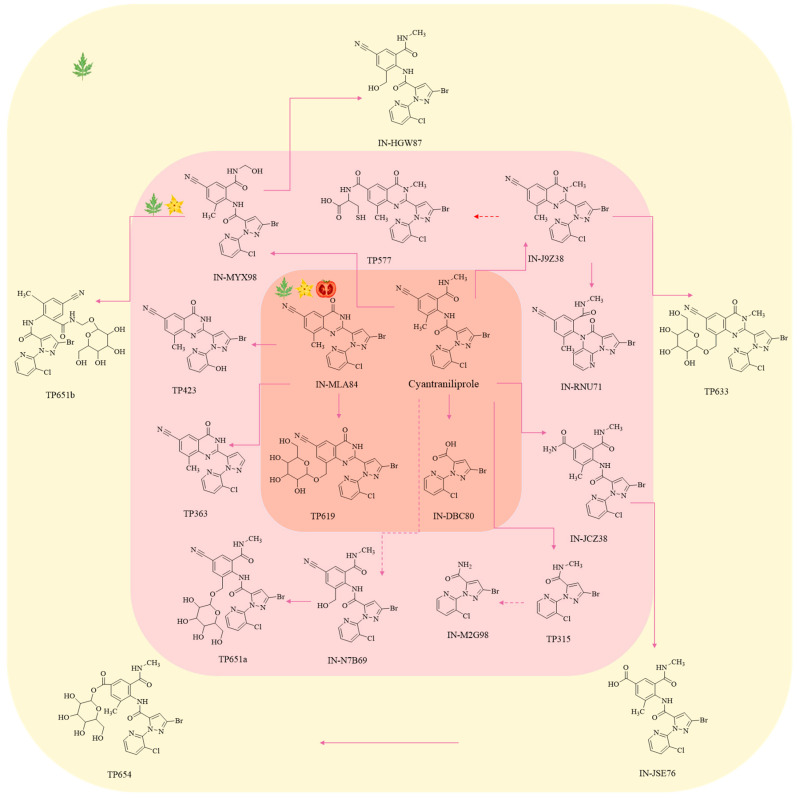
Distribution of cyantraniliprole metabolites in tomato. Arrows indicate the direction of degradation. Original illustration by the authors, based on data from references [110].

The metabolic transformation of cyantraniliprole within crops plays a central role in its environmental behavior, directly influencing both residue dynamics and dietary risk assessment. Studies in tomato plants, as a representative system, reveal that the metabolism of cyantraniliprole is tissue-specific. Leaves are the most metabolically active organs, with all 21 metabolites detected. Flowers contain 14 metabolites but at lower concentrations, while fruits contain only four metabolites, primarily IN-MLA84 and the degradation product IN-DBC80. The key intermediate IN-J9Z38 is not detected. Metabolism of cyantraniliprole in tomato is a multi-step, complex process occurring predominantly in aerial tissues, especially mature leaves. The metabolic network can be described as one major pathway accompanied by several minor branches. The primary metabolic pathway involves sequential steps in which cyantraniliprole first undergoes ring closure to form IN-J9Z38, then N-demethylation to yield IN-MLA84, and finally glycosylation to produce TP619. Minor metabolic routes include hydroxylation at specific sites (yielding IN-MYX98, etc.), dehalogenation of key intermediates (yielding IN-RNU71, etc.), glycosylation of other metabolites (yielding TP651, etc.), and cleavage of the amide bridge to form products such as IN-DBC80 [110]. These secondary metabolites are generally present at low levels. Although multiple metabolites are formed via oxidation, dealkylation, dehalogenation, glycosylation, and degradation reactions, the parent compound remains the predominant residue in all tissues until fruit maturity, indicating its relative stability and resistance to rapid, complete metabolism within the plant [110]. Therefore, from a dietary-risk perspective, risk assessment should focus primarily on the residue levels of the cyantraniliprole parent compound itself. The distribution of cyantraniliprole metabolites in tomato is shown in Figure 8.

### 4.3. Impact on Plant Physiological and Biochemical Processes in Plants

The translocation and metabolism of cyantraniliprole in plants might be accompanied by interference with their physiological and biochemical states. Research indicated that this compound and its analogs could interact with key antioxidant enzyme systems in plants.

**Figure 9:**
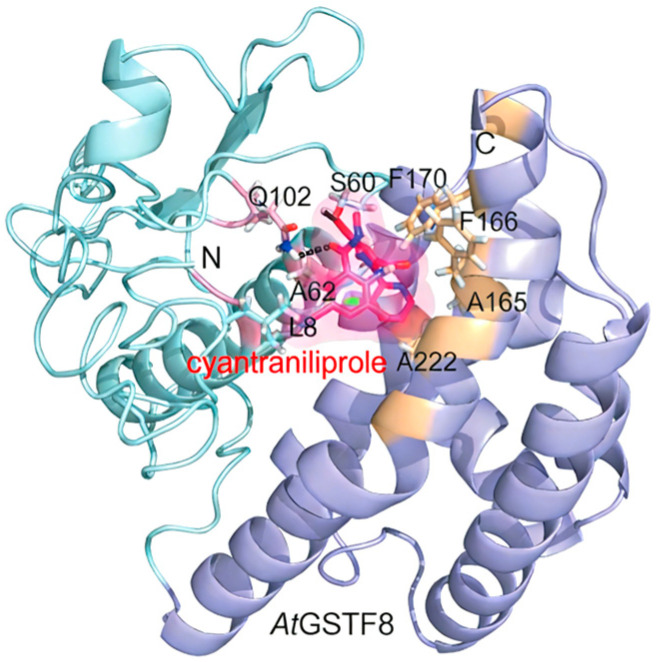
Predicted binding model of the interaction between cyantraniliprole and *AtGSTF8* [85]. Cyantraniliprole is shown in bright pink. The N-terminal domain of *AtGSTF8* is colored light cyan, and the C-terminal domain is shown in bright blue. The G-site and H-site are highlighted in bright pink and light yellow, respectively. Hydrogen bonds are represented by dashed lines. Residues F170, A165, L8, A222, F166, and A62 correspond to Phe170, Ala165, Leu8, Ala222, Phe166, and Ala62, respectively.

In plants, glutathione S-transferases (GSTs) constitute an important class of multifunctional antioxidant enzymes. Among them, the Phi class (e.g., *AtGSTF8*) was considered a key responder to chemical stress due to its abundance and substrate specificity [113,114,115,116]. For example, cyantraniliprole and the related insecticide chlorantraniliprole could affect the structure and function of the glutathione S-transferase Phi class (*GSTF1*) in common wheat (*Triticum aestivum*), leading to a significant reduction in its enzymatic activity [117]. In *Arabidopsis thaliana*, treatment with cyantraniliprole not only affected seedling growth but also specifically induced the upregulation of the *AtGSTF8* gene while simultaneously significantly inhibiting the glutathione S-transferase activity of its encoded protein (*AtGSTF8*) [85]. These enzymes typically consisted of two conserved domains: an N-terminal domain containing the glutathione-binding site (G-site) and a C-terminal domain forming the hydrophobic substrate-binding site (H-site) [86,118,119]. Molecular docking predictions suggested that the binding of cyantraniliprole to *AtGSTF8* might be primarily driven by hydrophobic interactions mediated by H-site residues (such as Phe170, Ala165, Ala222, and Phe166) [85] (Figure 9). This interaction might interfere with the normal function of GST enzymes, thereby affecting the antioxidant defense system of the plant.

### 4.4. Residue and Dissipation Dynamics in Soil, Water, and Crops

Cyantraniliprole has an established Acceptable Daily Intake (ADI) of 0.03 mg/kg body weight [117]. Its residual behavior and degradation in environmental and crop systems have been extensively studied in various matrices.

The degradation half-life of cyantraniliprole varied significantly among different crops and soil types, highlighting the substantial influence of crop species and environmental conditions on its persistence. For instance, its half-life ranged from 9.2 to 11.2 days in peppers but was generally shorter in cucumbers, tomatoes, pakchoi, and scallions, with a range of 1.3 to 6.4 days [13,14,120]. In soil, the half-life showed a wider variation from 2.6 to 20.8 days, potentially attributable to factors such as soil texture, climate, and microbial activity [13,14,120,121]. Its major metabolite, J9Z38, could be detected in some soils, but at low residue levels and without a clear dissipation pattern [121].

Field monitoring data under recommended application rates suggested a low residual risk for cyantraniliprole in agricultural produce and soil. In citrus orchards, residue levels in fruits and soil fell below the maximum residue limit (MRL = 0.7 mg/kg) one day after application. Soil residues typically dropped below the limit of quantification (0.01 mg/kg) within ten days [122].

Cyantraniliprole residues in vegetables could be effectively reduced through washing. A comparative study on spinach showed that soaking in water removed 19.6% of residues, and rinsing under running water removed 15.1%. The most effective removal (42.9%) was achieved by combining soaking with a neutral detergent [123].

The dissipation kinetics of cyantraniliprole in soil and crops followed an exponential model (C = Ae^Bt^). This rapid degradation pattern was evidenced by its short half-lives of 1.3–2.5 days in scallions and 2.6–4.3 days in soil, further supporting its classification as an easily degradable compound [120].

### 4.5. Environmental Persistence and Toxicity of Metabolites J9Z38

The environmental risk assessment of cyantraniliprole should also consider the persistence and toxicity of its key metabolite, J9Z38. Available data indicate that J9Z38 is more persistent in the environment than the parent compound, with a soil half-life ranging from 77 to 220 days. Consequently, J9Z38 is recognized as a toxicologically relevant transformation product, and its sustained persistence implies a significant potential for long-term residues and pollution [83,84]. Toxicity studies confirm that both J9Z38 and the parent compound can adversely affect soil organisms. For example, at an exposure concentration of 5.0 mg/kg, J9Z38 induces oxidative stress and cellular damage in earthworms, though the resulting oxidative injury is less severe than that caused by cyantraniliprole itself [88,124]. Importantly, the formation and accumulation of J9Z38 are closely tied to the dissipation of the parent compound. In pepper-cropping systems, for instance, the degradation half-life of cyantraniliprole is 9.2–11.2 days, while J9Z38 formed in field soil exhibits a half-life of 9.4 days [125]. In summary, a comprehensive environmental risk assessment for cyantraniliprole should integrate both its relatively rapid dissipation kinetics (e.g., half-lives of 1.3–6.4 days in crops such as cucumber and tomato [13,14,120], and 2.6–20.8 days in most soils [13,14,120,121]) and the high persistence of its metabolite J9Z38. Therefore, the potential long-term ecotoxicological effects of J9Z38 require continuous monitoring and should be integrated into risk-management plans.

## 5. Application Optimization and Risk Management

### 5.1. Innovative Formulation Technologies for Cyantraniliprole

Several innovative formulation technologies have been developed to improve the delivery and performance of cyantraniliprole.

Cyantraniliprole nano-formulations have been developed using polylactic acid and calcium carbonate. For both materials, particle size correlates with release rate: larger microspheres exhibit faster release, while smaller ones provide sustained release. All formulations display an initial burst release followed by a sustained release phase, with the active ingredient dispersed within the microspheres or adsorbed on their surface [126].

In addition to nano-formulations, recent studies have explored other innovative formulation technologies for cyantraniliprole. Dos Reis et al., developed cyantraniliprole-hybrid polymeric membranes and demonstrated their enhanced efficacy against the coffee leaf miner *Leucoptera coffeella* on coffee leaves. This approach represents a promising direction for improving the delivery and performance of cyantraniliprole in specific crop systems [127].

### 5.2. Cyantraniliprole Combined Applications

Since its patent expired in 2024, combination products containing cyantraniliprole have accelerated, including mixtures with insecticides of different modes of action (neonicotinoids, insect growth regulators, spinosad, abamectin, emamectin benzoate, chlorfenapyr), spray adjuvants, and biological control agents.

Spray adjuvants such as SC108 showed synergistic effects, reducing cyantraniliprole application by 20% while increasing efficacy by 7.6–37.4% and 5.5–34.1%, respectively [128]. Entomopathogenic nematodes combined with cyantraniliprole effectively controlled striped flea beetle while reducing chemical use [129]. The entomopathogenic fungus *Cordyceps fumosorosea* shows good compatibility with cyantraniliprole, with low toxicity to the parasitoid wasp *Tamarixia radiata* [70].

A recent study by Warsi et al., evaluated cyantraniliprole combined with entomopathogenic fungi (*Beauveria bassiana* and *Cordyceps javanica*) against *B. tabaci* across eight squash cultivars. Combined applications resulted in higher nymphal mortality than either agent alone. However, interactions varied by cultivar: additive effects were observed on some cultivars, while antagonistic effects occurred on others, highlighting the importance of cultivar-specific responses in optimizing control strategies [130].

### 5.3. Risk Mitigation and Management Recommendations

Currently registered formulations of cyantraniliprole include oil-based suspension concentrates, suspension concentrates, and seed treatment suspensions. These conventional formulations pose certain toxicity risks to non-target organisms. Microencapsulation technology, which protects active ingredients through coating materials, can significantly reduce these toxic effects. Cyantraniliprole microcapsules exhibit improved photostability and provide long-lasting efficacy against *S. frugiperda*, while enhancing safety to non-target organisms such as silkworms, honeybees, zebrafish, and earthworms compared to the technical material, thereby broadening its application scope [91].

Cyantraniliprole poses non-negligible toxicity risks to non-target organisms, particularly aquatic and soil-dwelling species. The environmental persistence of both the parent compound and its metabolites further exacerbates these risks. Effective risk management requires a multifaceted approach that includes optimizing application methods (e.g., soil treatment or seed treatment), strengthening environmental monitoring (particularly for the metabolite J9Z38), and exercising caution in sensitive ecosystems, such as rice–crayfish co-culture systems. Advancing fundamental research on its environmental fate, metabolic pathways, and chronic toxicity mechanisms is crucial for supporting accurate risk assessment.

Compared to foliar spraying, soil treatment, or seed treatment with cyantraniliprole may offer comparable or superior efficacy while reducing direct exposure to above-ground pollinators and natural enemies, and may extend the duration of effectiveness [131,132,133]. However, the persistence of the compound in soil may lead to prolonged uptake by crops, potentially resulting in bioaccumulation within plant tissues and increased selection pressure for resistance development [20]. Currently, knowledge regarding the metabolic pathways and distribution patterns of cyantraniliprole residues and metabolites in crops under long-term exposure scenarios remains limited [108].

## 6. Conclusions and Prospects

As a next-generation diamide insecticide, cyantraniliprole exhibits high efficacy due to its specific action on insect RyRs. However, long-term use is challenged by target-site mutations and enhanced metabolic detoxification in insects. Its high toxicity and environmental persistence pose significant risks to non-target organisms. To ensure sustainable use, a systematic resistance-monitoring system must be established, and a simple rotation with the same mechanism of action as diamides should be avoided. Risk mitigation should focus on formulation innovations (e.g., microencapsulation) and optimized application methods (e.g., seed treatment). Finally, advancing research on environmental behavior and chronic toxicity is essential to balance agricultural productivity with ecological security.

## Data Availability

No new data were created or analyzed in this study. Data sharing is not applicable to this article.
